# HyperHazeOff: Hyperspectral Remote Sensing Image Dehazing Benchmark

**DOI:** 10.3390/jimaging11120422

**Published:** 2025-11-26

**Authors:** Artem Nikonorov, Dmitry Sidorchuk, Nikita Odinets, Vladislav Volkov, Anastasia Sarycheva, Ekaterina Dudenko, Mikhail Zhidkov, Dmitry Nikolaev

**Affiliations:** 1 Samara National Research University, Moskovskoye Shosse 34, 443086 Samara, Russia; 2 Institute for Information Transmission Problems, the Russian Academy of Sciences, 127051 Moscow, Russia; odinets.ns@vis.iitp.ru (N.O.);; 3 Federal Research Center Computer Science and Control, the Russian Academy of Sciences, 119333 Moscow, Russia; 4 Smart Engines Service LLC, 117312 Moscow, Russia

**Keywords:** hyperspectral image (HSI), remote sensing, dehazing benchmark, real-world dehazing, agricultural field delineation, land classification

## Abstract

Hyperspectral remote sensing images (HSIs) provide invaluable information for environmental and agricultural monitoring, yet they are often degraded by atmospheric haze, which distorts spatial and spectral content and hinders downstream analysis. Progress in hyperspectral dehazing has been limited by the absence of paired real-haze benchmarks; most prior studies rely on synthetic haze or unpaired data, restricting fair evaluation and generalization. We present HyperHazeOff, the first comprehensive benchmark for hyperspectral dehazing that unifies data, tasks, and evaluation protocols. It comprises (i) RRealHyperPDID, 110 scenes with paired real-haze and haze-free HSIs (plus RGB images), and (ii) RSyntHyperPDID, 2616 paired samples generated using a physically grounded haze formation model. The benchmark also provides agricultural field delineation and land classification annotations for downstream task quality assessment, standardized train/validation/test splits, preprocessing pipelines, baseline implementations, pretrained weights, and evaluation tools. Across six state-of-the-art methods (three RGB-based and three HSI-specific), we find that hyperspectral models trained on the widely used HyperDehazing dataset fail to generalize to real haze, while training on RSyntHyperPDID enables significant real-haze restoration by AACNet. HyperHazeOff establishes reproducible baselines and is openly available to advance research in hyperspectral dehazing.

## 1. Introduction

Haze complicates image analysis and adversely affects the performance of machine vision systems [1,2]. The haze effect results from the partial absorption and scattering of light reflected from the scene as it travels through the turbid medium between the scene and the sensor. The goal of dehazing is to reconstruct the scene as it would appear under haze-free conditions, given a hazy observation. This study addresses the problem of remote sensing image dehazing (RSID). Following the formulation in [3], RSID methods can be categorized along two principal axes. The first distinguishes between mono-temporal approaches, which rely on a single hazy observation of the scene, and multi-temporal approaches [4], which exploit time-series data to recover haze-free information. The second axis differentiates mono-modal methods, which operate solely on optical imagery, from multi-modal methods that incorporate additional data sources such as Synthetic Aperture Radar (SAR) [3,5]. In this work, we focus on the mono-modal, mono-temporal setting.

Classical mono-temporal dehazing techniques are based on physical models of haze formation combined with simplifying assumptions, such as the dark channel prior (DCP), which assumes the existence of near-zero intensity values in local haze-free image patches [6], as well as other model-based approaches [7,8,9,10]. In recent years, deep learning methods have been introduced for dehazing, ranging from early convolutional neural networks (CNNs) such as FFANet [11], RSDehazeNet [12], and CANet [13] to transformer-based architectures like DehazeFormer [14], AIDTransformer [15], and RSDformer [16]. The latest Mamba-based models, such as RSDehamba [17] and HDMba [18], are reported to have improved capability to capture spatially varying haze distribution. Beyond model architectures, several works propose specialized training strategies to improve generalization, including multi-negative contrastive auxiliary learning [19].

Developing and evaluating dehazing algorithms requires datasets containing paired hazy and haze-free images of the same scene. However, haze is a difficult-to-reproduce and poorly controlled imaging condition. Open-source datasets with haze-free references and real haze generated by haze machines are available [20] for ground-based image dehazing, but this approach is infeasible for RSID. Only unpaired datasets with real haze had previously been available for RSID, and there was a lack of corresponding clean reference images [14]. To compensate for this, haze augmentation, where haze is synthetically applied to clear remote sensing images using physical or empirical models, became a common practice. However, the quality of the augmentation model critically affects the realism of the generated data. For example, [21] demonstrated that imperfections in the haze synthesis model can cause noticeable brightness loss in augmented images. Synthetic augmentation thus can only serve as a complementary tool and cannot fully replace real paired data [22]. The first dataset with hazy and haze-free remote sensing images containing RGB image pairs derived from multispectral (MS) satellite data, Real-World Remote Sensing Hazy Image Dataset (RRSHID), was introduced in 2025 [22].

Dehazing hyperspectral images (HSIs) presents unique challenges. Compared to RGB imagery, HSIs capture rich spectral information, offering considerable potential for downstream remote sensing tasks. While several specialized HSI dehazing methods have been proposed [11,15,18,23], evaluating their real-world performance remains difficult due to the absence of paired hyperspectral datasets with real haze. Unlike multispectral imagery, hyperspectral remote sensing data are expensive and technically complex to acquire. No dataset comparable to RRSHID currently exists for the hyperspectral domain. Moreover, there are no standardized haze-augmented benchmarks with defined train–validation–test splits or publicly available pre-trained models. The only available dataset, HyperDehazing [24], lacks standardized splits, while its accompanying repository [18] provides no trained weights. As a result, the capability of existing HSI dehazing models to generalize to real-world data remains largely unexplored.

To address this gap, we introduce the Remote Sensing Hyperspectral Dehazing Benchmark along with the Remote Sensing Real-World Hyperspectral Paired Dehazing Image Dataset (RRealHyperPDID)—the first hyperspectral dataset containing paired real-world hazy and haze-free remote sensing images.

The key contributions of this work are as follows:

- RRealHyperPDID, the first hyperspectral dataset containing paired real-world hazy and haze-free remote sensing images, providing the first opportunity to rigorously evaluate the real-world performance of hyperspectral dehazing algorithms.
- Agricultural field delineation and land classification annotations, enabling quantitative assessment of how dehazing influences downstream tasks relevant to precision agriculture and supporting downstream task quality assessment (DTQA) beyond traditional image-level metrics.
- Comprehensive evaluation of six dehazing algorithms, including both RGB-based and hyperspectral-specific methods, which reveals that existing state-of-the-art HSI models trained on previously available synthetic datasets (e.g., HyperDehazing) fail to generalize to real-haze data.
- RSyntHyperPDID, a large-scale synthetic hyperspectral dehazing dataset generated using a physically based haze formation model. Models trained on RSyntHyperPDID demonstrate effective generalization to real hazy data, establishing a new baseline.
- A fully open-source benchmark (HyperHazeOff), including standardized data splits (for both RSyntHyperPDID and HyperDehazing), preprocessing pipelines for hyperspectral and RGB data, baseline implementations, pretrained model weights, and evaluation scripts for image quality assessment (IQA) and DTQA—ensuring transparency and reproducibility for future research.

## 2. Related Work

### 2.1. Dehazing Datasets

The primary sources of HSIs used in RSID datasets are the Chinese Gaofen-5 (GF-5) satellite and the American AVIRIS airborne sensor. GF-5 data have a spatial resolution of 30 m per pixel, whereas the resolution of AVIRIS data depends on flight altitude and typically ranges from 2 to 20 m per pixel. Both sensors cover a similar spectral range (400–2500 nm), but the GF-5 provides higher spectral resolution—330 spectral channels compared to 224 channels for AVIRIS.

#### 2.1.1. Datasets with Synthetic Haze

Synthetic haze augmentation is commonly used to generate paired data for training and evaluating dehazing algorithms. In this approach, haze is artificially added to clear HS remote sensing images using physical models of atmospheric scattering, which simulate the wavelength-dependent attenuation and diffusion of light.

Commonly, one of two models is employed for synthetic haze augmentation. The atmospheric scattering (AS) model is physically based and accounts for both the additional illumination caused by light scattering from haze particles and the partial absorption of light reflected from the scene:

(1) $$I\left(x,\lambda \right)=J\left(x,\lambda \right)t\left(x,\lambda \right)+A\left(\lambda \right)\left(1-t\left(x,\lambda \right)\right),\;t\left(x,\lambda \right)=e^{-\beta (\lambda ,\gamma )d(x)},$$

where I(x,λ) is the hazy image, J(x,λ) is the haze-free image, A(λ) represents atmospheric light, t(x,λ) is the haze transmission map (HTM), β(λ,γ) is the scattering coefficient (dependent on atmospheric particle properties, including size), and d(x) is the scene depth or, in remote sensing, the depth of the haze at *x*.

A simplified alternative model is expressed as

(2) I(x,λ)=J(x,λ)+k(λ)t(x),

where k(λ) is the haze abundance coefficient, and the HTM t(x) is assumed to be wavelength(λ)-independent.

Model (2) has been employed in constructing the HyperDehazing [18] and HDD [25] datasets. The HDD dataset includes a single AVIRIS HS image with artificially added haze, and the corresponding augmentation procedure is described only briefly [25]. In contrast, the HyperDehazing dataset provides a detailed description of its haze synthesis process, known as Hazy HSI Synthesis (HHS) [18]. HyperDehazing contains 2000 pairs of GF-5 HS images with augmented haze. To generate the HTM, donor images containing real haze were used, and their transmission maps (t(x)) were extracted using a DCP-based method. A total of 20 distinct HTMs were derived. The haze abundance coefficient k(λ) was estimated for five representative land-cover types—vegetation, buildings, water, bare soil, and mountains—based on images with real haze.

The AS model has primarily been applied to MS imagery [12,14,26] through Channel Correlation-Based Haze Synthesis (CCHS). In CCHS, the HTM for each spectral channel $\lambda_{i}$ is derived from a guided channel $t_{g}\left(x\right)$ using the wavelength ratio:

(3) $$t_{\lambda_{i}}\left(x\right)=e^{{\left(\frac{\lambda_{g}}{\lambda_{i}}\right)}^{\gamma }lnt_{g}\left(x\right)}.$$

The guided-channel HTM $t_{g}\left(x\right)$ is typically extracted from real hazy images. In [26], the scattering coefficient β is considered spatially uniform. However, as noted in [12], remote sensing images can cover large geographical areas (up to tens of kilometers), leading to spatial variations in haze density and properties. Accordingly, the scattering coefficient is modeled as spatially dependent through a parameter γ(x), i.e., β(λ,γ(x)). γ(x) is estimated from $t_{g}\left(x\right)$. The atmospheric light A(λ) is estimated as the mean intensity of the top 0.01% brightest pixels in each spectral channel.

#### 2.1.2. Datasets with Real Haze

Both HyperDehazing and HDD also include subsets of HS images with real haze but without corresponding haze-free references. HyperDehazing contains 70 GF-5 images, while HDD includes 20 hazy GF-5 satellite images and 20 AVIRIS airborne images.

**Figure 1:**
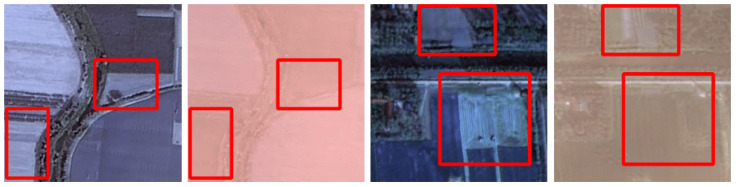
Examples from RRSHID [22] showing scenes with significant temporal changes (highlighted with red boxes) between paired hazy and haze-free images.

Paired hyperspectral datasets with real haze had not been available until recently. The first such dataset was proposed for RGB imagery—the RRSHID dataset [22]—which contains paired RGB images of the same region under hazy and haze-free conditions. The source multispectral data for RRSHID were obtained from the China High-Resolution Earth Observation System (CHEOS), and these multispectral images were then converted to RGB by the authors [22] to construct the RRSHID dataset. A key limitation of RRSHID is the presence of temporal scene changes between the paired images. Moreover, the dataset provides no metadata describing the nature or extent of these temporal changes, which complicates its use for objective evaluation. As described in [22], RRSHID image pairs were selected automatically based on acquisition time proximity (1–3 months apart). We can observe that this leads to notable differences in land surface appearance, especially in agricultural areas (see Figure 1).

### 2.2. Dehazing Quality Assessment

Evaluation of RSID methods can be performed using two main approaches. The first is image quality assessment (IQA), which evaluates the visual or spectral fidelity of the resulting dehazed images. The second is downstream task quality assessment (DTQA), which assesses how dehazing influences the performance of subsequent remote sensing tasks.

The IQA approach can be further categorized into Full-Reference IQA (FRIQA) and No-Reference IQA (NRIQA). FRIQA involves comparing a dehazed image with a corresponding clean reference image using universal image quality metrics. These include classical measures such as Root Mean Square Error (RMSE), Peak Signal-To-Noise Ratio (PSNR), Structural Similarity Index (SSIM) [27], and Spectral Angle Mapper (SAM) [28], as well as learned perceptual metrics such as Learned Perceptual Image Patch Similarity (LPIPS) [29]. In contrast, NRIQA is applied when reference images are not available (e.g., for unpaired datasets such as the real-haze subsets of the HyperDehazing and HDD datasets). In this case, the quality of a hazy or dehazed image is evaluated independently using neural network-based metrics designed specifically for dehazing assessment, such as FADE [30], BIQME [31], and VDA-DQA [32].

While DTQA is less frequently used for RSID evaluation, it provides valuable insight into the practical impact of dehazing on remote sensing applications. For example, Guo et al. [12] demonstrated through qualitative analysis of Normalized Difference Vegetation Index (NDVI) maps derived from MS imagery that haze significantly distorts vegetation information and that their proposed dehazing method partially restores these distortions. Li et al. [1] similarly used a haze synthesis model to analyze how varying haze densities affect classification accuracy. The most recent study by Zhu et al. [22] emphasizes the importance of constructing dehazing benchmark datasets with specific downstream task annotations as a direction for future work.

## 3. Proposed Remote Sensing Hyperspectral Datasets

This section introduces two datasets designed for evaluating RSID methods: RRealHyperPDID (Remote Sensing Real-World Hyperspectral Paired Dehazing Image Dataset) and RSyntHyperPDID (Remote Sensing Synthetic Hyperspectral Paired Dehazing Image Dataset). Both datasets are derived from the AVIRIS project database, which provides hyperspectral and RGB imagery acquired from aircraft. The images are approximately 1000 pixels wide, with lengths varying up to 30,000 pixels. To avoid confusion between the original images and the smaller cropped fragments used for analysis, these source images are hereafter referred to as flight-line images.

### 3.1. RRealHyperPDID: Remote Sensing Real-World Hyperspectral Paired Dehazing Image Dataset

During the operation of the AVIRIS project, thousands of flight-line images have been collected and made publicly available, some of which capture the same surface areas at different times. In several cases, one image of the same area contains haze, while another remains clear. This observation enabled the creation of a dataset consisting of real-world hyperspectral image pairs—captured under hazy and haze-free conditions.

To construct the dataset, the following procedure was used to identify suitable flight-line image pairs:

- Flight-line images annotated with haze in the AVIRIS Data Table were initially selected.
- RGB visualizations of these HSIs were downloaded and visually inspected to confirm the presence of haze.
- Using the interactive map provided by the AVIRIS Data Portal, hazy images with corresponding clear counterparts were identified and selected.

**Figure 2:**
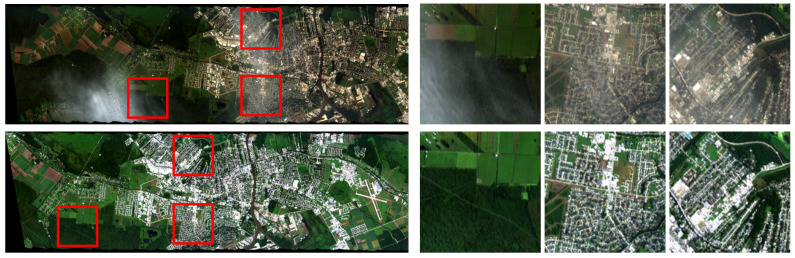
A set of flight-line images consisting of one hazy and one clear image, along with three pairs of manually annotated rectangular areas affected by haze on the hazy flight-line image (highlighted with red boxes).

As a result, 17 distinct zones containing multiple flight-line images were identified, each including one hazy image. An example of such image pairs from the same zone is shown in Figure 2.

**Figure 3:**
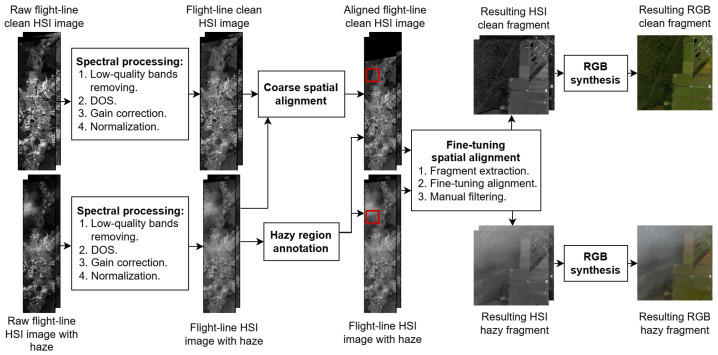
Pipeline for deriving the RRealHyperPDID dataset from raw AVIRIS flight-line images.

The complete processing pipeline used to construct the RRealHyperPDID dataset is shown in Figure 3.

#### 3.1.1. Spectral Processing

Each selected flight-line image I(x,y,λ) underwent the following spectral processing steps:

1. Removal of low-quality bands: bands 104–116, 152–170, and 214–224 were excluded, following standard preprocessing [33].
2. Dark Object Subtraction (DOS):
  $\forall \lambda_{i}$: $I_{DOS}\left(x,y,\lambda_{i}\right)=I\left(x,y,\lambda_{i}\right)-\min_{x,y}\left(I\left(x,y,\lambda_{i}\right)\right)$.
3. Gain correction:
  $I_{Gain}\left(x,y,\lambda \right)=I_{DOS}\left(x,y,\lambda \right)/gain\left(\lambda \right)$, where gain(λ)=300, λ∈(365,1392), gain(λ)=600, λ∈(1392,1867), gain(λ)=1200, λ∈(1867,2497).
4. Normalization: pixel values were normalized with the global maximum across all bands, max(I(x,y,λ)).

DOS is a standard atmospheric correction method that alters inter-band ratios by removing distortions induced by atmospheric transmission. Gain correction is another standard procedure that also modifies digital number values differently across bands; however, its objective is to restore radiometric values, modified to store images with the int16 format. Finally, normalization preserves inter-band ratios while mapping pixel values to a consistent range, which is necessary for subsequent processing (e.g., dehazing neural networks typically require inputs scaled to [0,1]).

#### 3.1.2. Spatial Alignment

**Figure 4:**
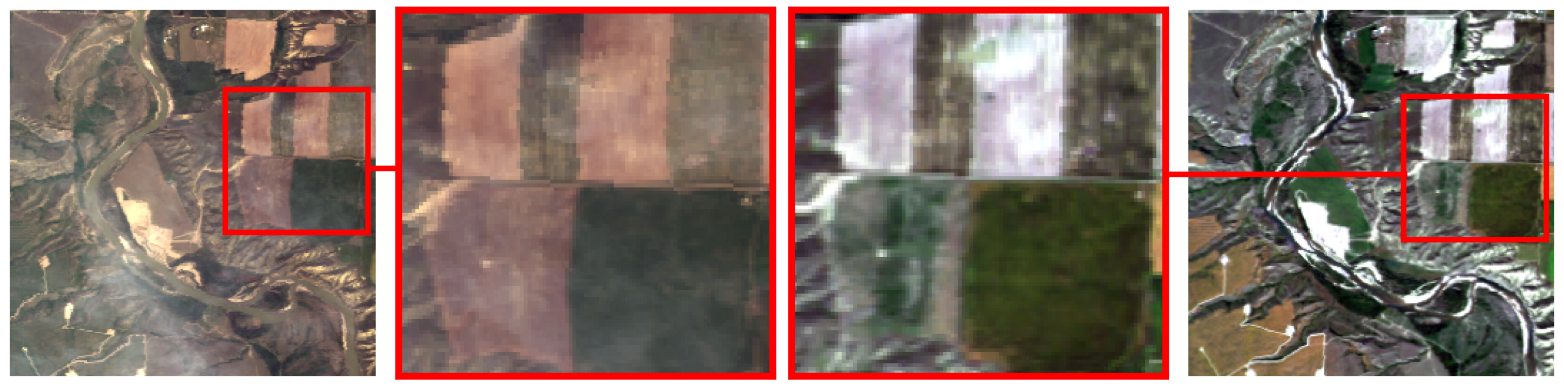
An example of geometric distortions in AVIRIS data.

The AVIRIS data are georeferenced; however, the positional accuracy of the selected flight-line images was insufficient for precise alignment of fragments within our dataset, with errors reaching up to 10 pixels. In addition, several scenes exhibited geometric distortions that could not be corrected using standard alignment techniques (see Figure 4).

To ensure accurate co-registration, the following alignment procedure was applied:

1. Automatic coarse alignment of flight-line images using the SIFT (Scale-Invariant Feature Transform) [34], followed by estimation of projective transformation parameters via RANSAC (Random Sample Consensus) [35]. Feature points were detected on the full flight-line images.
2. Hazy region annotation: In the selected flight-line images, haze is typically localized rather than uniformly distributed. To capture affected areas, rectangular patches 256×256 pixels in size containing visible haze were manually annotated in each hazy flight-line image. The corresponding patches were then extracted from the matching haze-free images using the same spatial coordinates (see Figure 2).
3. Alignment fine-tuning using feature points detected only inside processed fragments.
4. Final expert verification and exclusion of pairs where distortions could not be adequately compensated (as in Figure 4).

#### 3.1.3. RGB Synthesis

In addition to HSI data, our dataset includes an RGB version of each image generated through color synthesis from the HSIs. Two color synthesis methods were applied: The first is Color Synthesis Narrow Channels (CSNC) based on the AVIRIS quicklook generation method but without final JPEG compression. Three narrow spectral channels were extracted: R =646 nm, G =547 nm, B =449 nm. The second is Color Synthesis Standard Observer (CSSO) [36] based on the CIE Standard Observer color matching functions. Weighted spectral integration of the HS signal was performed at each pixel.

#### 3.1.4. Subset of Pairs with Closest Scene Conditions

**Figure 5:**
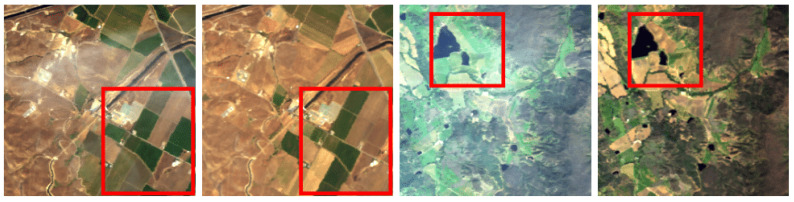
Examples of RRealHyperPDID images showing the same area that has changed significantly between acquisitions taken just 19 days apart (18 April 2014 and 7 May 2014).

An additional criterion was introduced to identify the most suitable image pairs for evaluation. All other imaging conditions should remain as constant as possible [37] to enable full-reference metrics to accurately assess the degree of haze removal. The primary sources of variation are illumination changes and alterations in the Earth’s surface objects (see Figure 5).

Selecting images based solely on temporal proximity is insufficient to guarantee scene similarity. As discussed in Section 2.1, the spectral reflectance of certain land-cover types, such as agricultural fields, may change significantly over short time intervals (see Figure 1). Expert evaluation was therefore conducted to identify a subset of image pairs where hazy and haze-free scenes were most similar in all aspects other than the presence of haze. To ensure the reproducibility of the results, we have published the outcome of the expert-based selection of the most similar scenes as a separate list of images in the benchmark repository.

#### 3.1.5. Dataset Characteristics

The proposed dataset contains 110 hazy fragments 256×256 pixels in size. Each hazy fragment is associated with at least one haze-free reference, with some having multiple references, resulting in 161 paired samples in total. Each hyperspectral image is accompanied by two RGB versions—one generated using the CSNC method and the other using CSSO.

The acquisition dates of the selected images range from 15 August 2011 to 2 April 2021, covering diverse terrain types, including mountainous, coastal, urban, and suburban regions. The spatial resolution varies from 8 to 18 m per pixel, depending on flight altitude. After removing low-quality bands, each image contains 182 spectral bands, spanning 365–2396 nm.

A subset of 21 image pairs (hazy vs. clear) was identified as being the most visually consistent across all parameters except for the presence of haze. Of these, 14 pairs were acquired within less than a month of each other, while 7 pairs had acquisition gaps ranging from 11 months to 1 year.

### 3.2. RSyntHyperPDID: Remote Sensing Synthetic Hyperspectral Paired Dehazing Image Dataset

We created a synthetic dataset, RSyntHyperPDID, based on the AVIRIS database. The dataset is sufficiently large to serve as a training set for dehazing models. Each RSyntHyperPDID image has a size of 256×256 pixels, identical to that of RRealHyperPDID. We used a modified version of the CCHS method described in Section 2.1.1 for haze augmentation of the HSI data. Given the small fragment size and the high spatial resolution of AVIRIS, we employed a version of the method with a spatially independent γ parameter [26].

**Figure 6:**
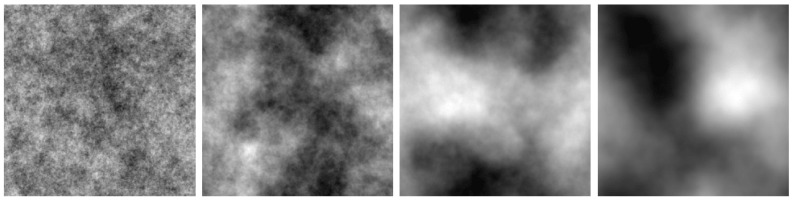
Examples of randomly generated maps with parameters ψ={2.0,3.0,4.0,5.0}.

The proposed modification to the CCHS method concerns the generation of the HTM t(x). Accurately extracting haze from real hazy images requires careful manual preparation: not every image contains haze, and even among those that do, extracted haze may also contain contrasting surface elements. Consequently, very few HTMs are typically used in data generation; for instance, Ref. [18] reports using only 20 distinct HTMs for the 2000 dataset images. To overcome this limitation, instead of extracting haze from real images, we randomly generated t(x) as a two-dimensional random field parameterized by ψ, that allows control over the spatial uniformity of t(x) (see Figure 6). We used ψ={3.0,4.0} in our experiments. We used 218 initial haze-free fragments, each of which was processed with two unique randomly generated HTMs. Combining this with six gamma variations, γ={0.0,0.5,0.7,1.0,2.0,4.0} [12], resulted in 12 distinct hazed versions per fragment.

A comparison of the characteristics of the proposed datasets with other known RSID datasets is provided in Table 1.

### 3.3. Downstream Task Quality Assessment

To extend the applicability of the HyperHazeOff benchmark beyond image quality assessment (IQA), we provide downstream task quality assessment (DTQA).

#### Delineation-Based Dehazing Quality Assessment

First, we evaluated the task of agricultural field delineation, which is related to segmentation but focuses on accurately detecting cultivated field boundaries relative to uncultivated land [38]. This task is essential for maintaining up-to-date cadastral maps and monitoring agricultural activities.

From the 110 scenes in RRealHyperPDID, 34 scenes containing agricultural fields were selected, corresponding to 86 clear and hazy fragments (in some cases, multiple clear fragments corresponded to a single hazy scene). In total, 3623 fields were manually delineated by experts using CSNC RGB images.

**Figure 7:**
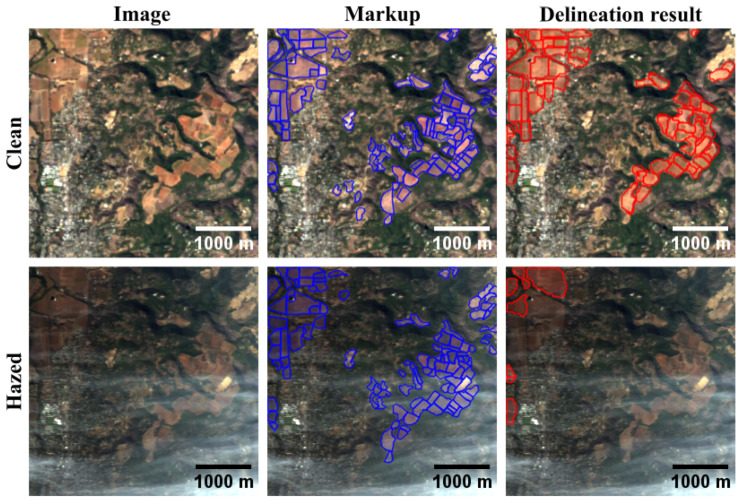
Results of applying the DA agricultural field delineation method to clean and hazy RGB images from the RRealHyperPDID dataset generated using the CSNC method. The blue delineation mask represents manual annotations, while the red delineation mask corresponds to the algorithm’s output.

The Delineate Anything (DA) method [39]—a state-of-the-art neural network approach—was employed for automatic delineation. The method’s pretrained model weights are publicly available, and its resolution-agnostic design allows for direct application to our RGB data without additional tuning. An example of DA’s performance on clear and hazy images is shown in Figure 7: the presence of haze notably reduces delineation quality.

To quantify performance, we used both the DICE coefficient and the object-based $DICE_{obj}$ metric [38]. While the pixel-based DICE metric is standard for segmentation evaluation, it is relatively insensitive to over- and under-segmentation errors, while $DICE_{obj}$ provides a more robust assessment of delineation accuracy. $DICE_{obj}$ is defined as

(4) $$DICE_{obj}=\frac{2\times N_{one2one}}{N_{m}+N_{ref}}\times 100\%,$$

where $N_{one2one}$ is the number of uniquely matched fields, $N_{m}$ is the number of fields detected by the evaluated method, and $N_{ref}$ is the number of reference fields. Two fields $f_{ref}^{i}$ and $f_{m}^{j}$ are considered uniquely matched if their Jaccard index satisfies $J(f_{ref}^{i},f_{m}^{j})>0.5$, and no other fields $f_{ref}^{k}$ and $f_{m}^{l}$ exist such that $J(f_{ref}^{k},f_{m}^{j})>0.5$ or $J(f_{ref}^{i},f_{m}^{l})>0.5$, respectively.

A comparison of DA method performance on clear and hazy fragments is presented in Table 2. As expected, delineation accuracy on hazy images is consistently lower than on haze-free ones.

### 3.4. Land Classification Quality Assessment

The previous section presented DTQA for RGB dehazing. Here, we extend DTQA to MS data by evaluating the impact of haze on land use and land cover (LULC) classification [40]. LULC classification is performed at the image (or image-fragment) level rather than per pixel, making it less sensitive to local distortions and label noise. To this end, we selected the neural network-based method proposed by Chong [41] for inclusion in the benchmark. This method is open source, provides a complete training pipeline, and achieves performance comparable to other neural network approaches (e.g., [42]).

We reproduced the training procedure using the EuroSAT dataset [40], which contains multispectral Sentinel-2 image patches labeled into ten classes: SeaLake, Forest, HerbaceousVegetation, PermanentCrop, Industrial, AnnualCrop, River, Pasture, Highway, and Residential. Following [41], we used 5000 fragments for pretraining. We additionally constructed a training set based on our benchmark HSI data. MS synthesis was performed using the Sentinel spectral sensitivity functions. Expert annotation was conducted for our MS fragments. The training set contained 852 clean MS fragments derived from haze-free images in RSyntHyperPDID. The test set comprised 666 clean and 421 hazy MS fragments extracted from RRealHyperPDID.

After initial pretraining on EuroSAT using transfer learning, the model was fine-tuned on our dataset using progressive unfreezing and differential learning rates. This approach enables adaptation to our data while retaining generalizable features learned from the larger dataset. The classification accuracy was 74.56% for clean fragments and 56.29% for hazy fragments, demonstrating that haze significantly degrades LULC performance.

## 4. Dehazing Evaluation

This section presents three sets of experiments comparing state-of-the-art dehazing methods: FRIQA-based evaluation of RGB dehazing methods using RGB images from the RRealHyperPDID dataset; and FRIQA-based evaluation of hyperspectral dehazing methods; DTQA-based evaluation, assessing the effect of RGB and HS dehazing on agricultural field delineation and land classification performance. The RRealHyperPDID dataset incorporates cases where a single hazy image corresponds to multiple clean reference images that differ in their illumination conditions. As a result, there is no unique ground-truth representation of the haze-free scene. We therefore consider three strategies for handling multiple reference images per hazy observation:

1. Best-value selection: The score is computed against all available reference images for each dehazed image and each metric, and the best score is retained. This produces a single representative metric value per scene, although the selected reference may differ across metrics.
2. Fixed-reference evaluation: One reference image is selected for each scene and used consistently across all evaluated methods and metrics. Metrics are computed only for the fixed hazy/dehazed–reference pairs and then averaged across all scenes.
3. Reference-averaged evaluation: For each scene, metrics are computed for all hazy/dehazed–reference combinations, averaged over references, and then averaged across scenes.

From the perspective of dehazing, the haze-free images with varying illumination are all equally valid results, and the evaluation should not penalize one in favor of another. Although correcting for illumination to restore the reflectance coefficients could help mitigate this issue, HSI illumination correction is a complex problem that lies beyond the scope of this work. Selecting a single reference introduces bias toward that particular acquisition, whereas averaging across references may obscure meaningful differences between methods. The best-value selection strategy offers a more practical assessment: the goal of dehazing is not to reproduce a specific reference image but to recover a plausible haze-free scene. While we consider the best-value selection strategy to be the most appropriate for remote sensing dehazing and therefore report metrics based on it in this paper, all three evaluation strategies are included in the benchmark for completeness and reproducibility. Notably, the relative ranking of the evaluated state-of-the-art dehazing methods remains consistent across all experiments regardless of the reference-handling strategy used.

### 4.1. RGB Dehazing Image Quality Assessment Results

We evaluated three RGB dehazing methods: DCP, the classical Dark Channel Prior method [6]; Color Attenuation Dark Channel Prior (CADCP), a recent approach [10] that effectively compensates for color haze, simultaneously estimating haze density from the DCP and ambient illumination from the Color Attenuation Prior (CAP), where the luminance–saturation of each pixel is linearly dependent on scene depth or haze concentration [9]; and DehazeFormer, a modern neural network-based method [14] with publicly available pretrained weights. We employed three classical metrics—PSNR, SSIM, and SAM—along with two perceptual neural metrics—LPIPS and DISTS [43]. To assess chromaticity difference, we used the specialized metric $CD_{proLab}$ [44].

**Figure 8:**
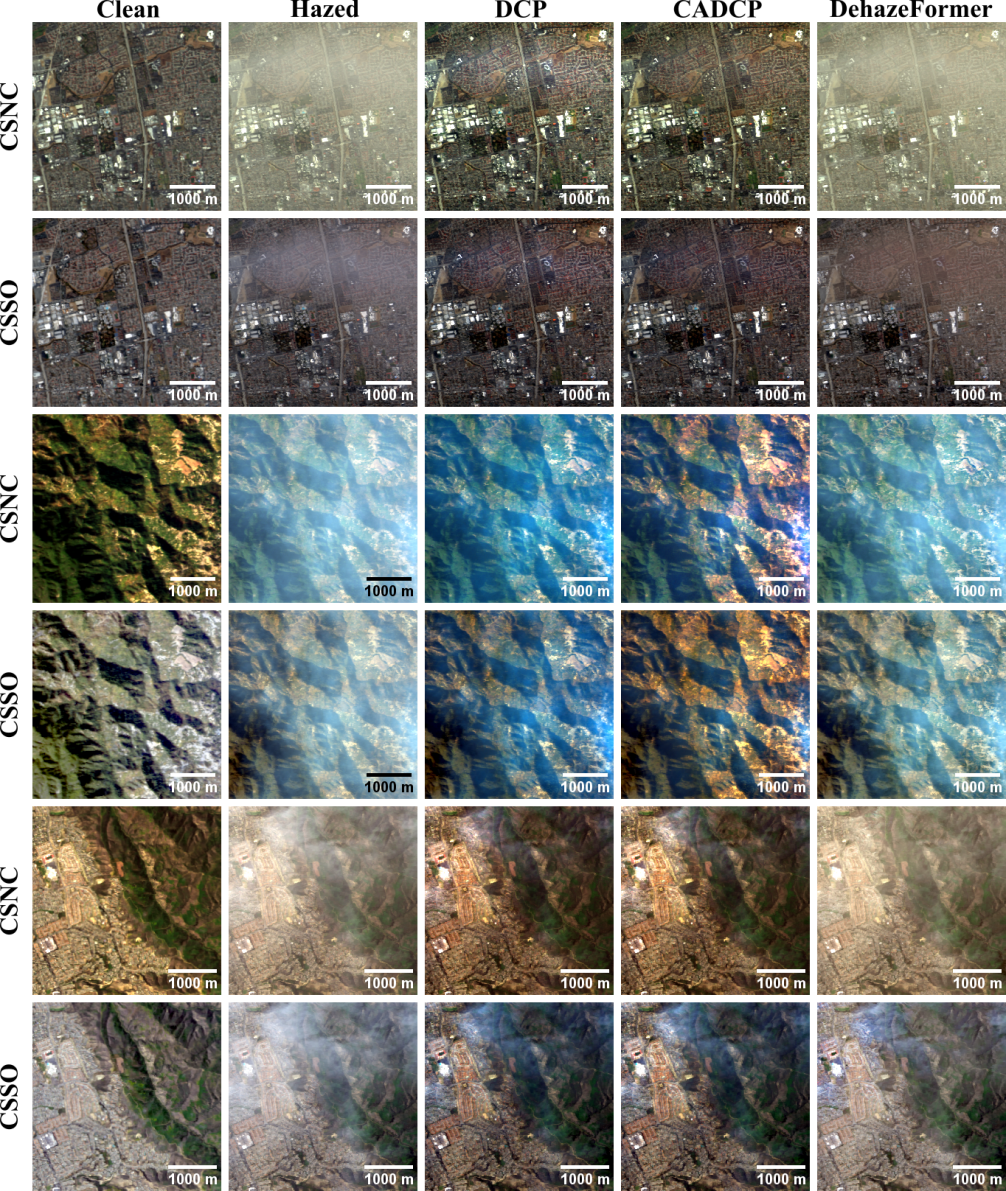
Examples of RGB dehazing results obtained using the CSNC and CSSO RGB synthesis methods.

The results of the RGB dehazing methods applied to the CSNC and CSSO outputs are presented in Table 3 and Table 4 and illustrated in Figure 8. In the case of RGB images synthesized with the CSNC method, all dehazing methods generally improve the visual quality of hazy images, with the exception of the SAM metric, according to which only DehazeFormer achieves improvement. Since the mean values of several methods were close and exhibited substantial overlap in their confidence intervals, a paired *t*-test was performed. The test revealed that, for the SSIM and LPIPS metrics, the apparent superiority of CADCP over DehazeFormer is not statistically significant at the α=0.05 level. Additionally, no significant difference was observed between DCP and DehazeFormer for the DISTS metric. Overall, CADCP and DehazeFormer demonstrate the best performance among the evaluated approaches. However, while CADCP effectively removes haze, it introduces noticeable chromatic distortions, as reflected by its higher $CD_{proLab}$ values.

Regarding RGB images generated with the CSSO method, only DehazeFormer achieves statistically significant improvement over hazy inputs for PSNR, DISTS and LPIPS. In contrast, SAM and $CD_{proLab}$ exhibit the best values for the unprocessed (hazy) images—an inconsistency with qualitative observations. As shown in Figure 8, CADCP and DCP visibly reduce haze and, in some cases, even produce visually superior results to DehazeFormer.

To resolve discrepancies between quantitative metrics and perceptual quality, we conducted an expert evaluation involving four specialists from the Faculty of Space Research (Moscow State University) and the Vision Systems Laboratory (Institute for Information Transmission Problems). The experts performed pairwise comparisons among DCP, CADCP, DehazeFormer, and the original hazy images, selecting in each case the image with the least perceived haze or indicating “cannot choose”. The results of the pairwise comparisons were aggregated into a global score using the Bradley–Terry model [45]. The resulting expert-based scores are presented in Table 5. According to the expert evaluation, the methods rank in the following order of dehazing effectiveness for both RGB visualization types: CADCP; DCP; DehazeFormer; Hazed.

In the case of RGB images generated with the CSNC method, this expert-derived ranking is fully consistent with the PSNR metric, while for CSSO-RGB, none of the evaluated metrics show a reliable correlation with experts’ perception.

#### Evaluation on the Subset with the Closest Scenes

The discrepancy between the expert rankings and the metric-based rankings, as well as the resulting inadequacy of certain metrics, may be explained by the variation in scene content and imaging conditions, as discussed in Section 3.1.4. To clarify this issue, we compared the metric rankings separately for the subset of closely matched scenes and for the remaining scenes from the RRealHyperPDID dataset. Table 6 presents the Kendall correlation coefficients between the metric-based rankings of the dehazing algorithms and the reference expert rankings. For the results on the subsets, an additional paired statistical test was conducted. A dash in the table indicates that the metric did not produce a statistically significant ranking.

Regarding RGB images synthesized with the CSNC method, the metric rankings were consistent with the expert ranking for the subset of closest scenes, with the exception being SAM and LPIPS metrics. The expert rankings aligned with the PSNR metrics for the remaining scenes. In the case of RGB images synthesized with the CSSO method, the rankings for both subsets showed no significant correspondence with the expert evaluation.

### 4.2. HS Dehazing Image Quality Assessment Results

The hyperspectral dehazing methods selected for comparison were AACNet [23], HDMba [18], and AIDTransformer [15]. Two experiments were conducted. In the first experiment, the selected neural network models were trained on the HyperDehazing dataset following the procedures described in the corresponding publications and subsequently tested on the RRealHyperPDID dataset, which was spectrally harmonized to match the spectral resolution of HyperDehazing. In the second experiment, the same models were trained on the proposed RSyntHyperPDID dataset and also evaluated on RRealHyperPDID.

The standard image quality metrics (PSNR, SSIM, and SAM) were used for evaluation. It should be noted, however, that the SAM metric showed weak correlation with expert rankings for RGB data, even within the subset of closely matched scenes. In addition, the evaluation of HS results is inherently challenging: the data cannot be assessed visually (even by experts), and the lack of field delineation methods for hyperspectral imagery makes delineation-based DTQA evaluation infeasible.

#### 4.2.1. Experiment 1

Since pre-trained models from HyperDehazing were not publicly available, we reproduced the authors’ training procedure. As reported in [24], 1800 images were used for training and 200 for testing; however, no exact split was provided. All models were trained for 10,000 iterations with their original hyperparameter configurations. AACNet was trained using the ADAM optimizer ($\beta_{1}=0.9$, $\beta_{2}=0.99$, $\epsilon =10^{-8}$) with a batch size of 32, an initial learning rate of $2\times 10^{-4}$, and a Mean Squared Error (MSE) loss function. AIDTransformer was trained using the ADAM optimizer with an initial learning rate of $2\times 10^{-4}$, a cosine annealing learning rate schedule, and an $L_{1}$ loss function. HDMba was trained using the ADAM optimizer ($\beta_{1}=0.9$, $\beta_{2}=0.999$, $\epsilon =10^{-8}$) with a batch size of 4, an initial learning rate of $1\times 10^{-4}$, a cosine annealing schedule, and a combined loss function $\lambda_{1}\cdot \mathrm{MSE}+\lambda_{2}\cdot L_{1}$ with $\lambda_{1}=1$ and $\lambda_{2}=0.1$. All models were implemented in PyTorch (version 1.10.2) framework and trained on NVIDIA GPUs: GeForce RTX 2080 Ti for AACNet and AIDTransformer, and Quadro GV100 for HDMba. Training required 8.5 GPU h for AACNet (9 GB memory usage), 8.7 GPU hours for AIDTransformer (11 GB memory usage), and 8.2 GPU hours for HDMba (30 GB memory usage).

**Figure 9:**
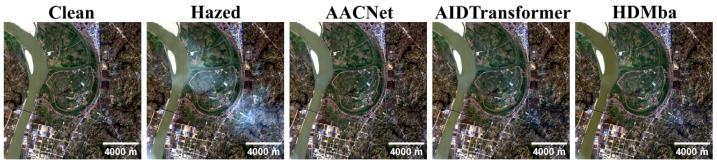
RGB visualization comparison of HS dehazing methods trained on the HyperDehazing synthetic training set and evaluated on the test set.

**Figure 10:**
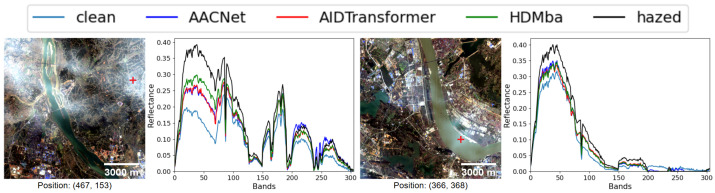
Spectral reconstruction performance for various HS dehazing methods on the HyperDehazing test set. The red plus sign indicates the pixel location for which the spectral reconstruction is shown.

The test results on the HyperDehazing dataset achieved quality metrics comparable to those reported in [18] (Table 7). Figure 9 shows that all methods effectively removed synthetic haze, while Figure 10 presents the spectral reconstruction at a representative pixel, demonstrating that the methods approximate the clean image spectra.

The number of channels and the spectral ranges differ between HyperDehazing and RRealHyperPDID. The problem in which the response of one sensor is used to predict the response of another sensor with the known spectral sensitivity to the same radiation is called spectral harmonization [46]. Sensor-response-based harmonization requires a set of aligned image pairs, where the same area is captured by both sensors involved, for parameter tuning. Since such a dataset was unavailable in our case, we adopted linear interpolation to the RealHyperPDID images to match HyperDehazing spectral configuration.

**Figure 11:**
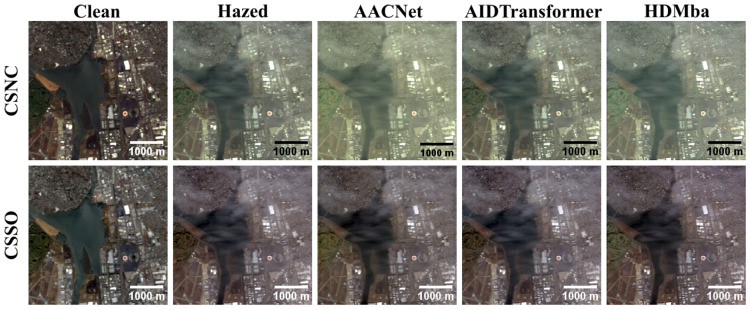
RGB visualization comparison of HS dehazing methods trained on the HyperDehazing synthetic dataset and tested on the RRealHyperPDID real-world dataset.

**Figure 12:**
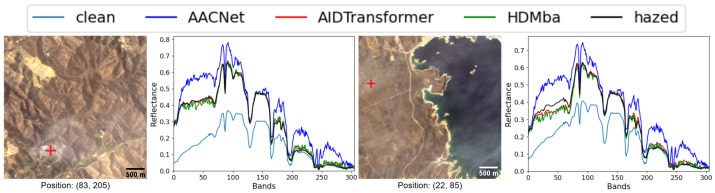
Spectral reconstruction performance of HS dehazing methods on the RRealHyperPDID dataset. The red plus sign indicates the pixel location for which the spectral reconstruction is shown.

The metric-based results are presented in Table 8. None of the HS dehazing methods produced a clear improvement over the original hazed images. This conclusion is also supported by qualitative analysis: Figure 11 shows RGB visualizations of the dehazed results, and Figure 12 presents spectral profiles at selected points.

#### 4.2.2. Experiment 2

The RSyntHyperPDID dataset was divided into training and validation subsets containing 2354 and 262 fragments, respectively. The training hyperparameters were identical to those used for the HyperDehazing dataset (see the previous section).

**Figure 13:**
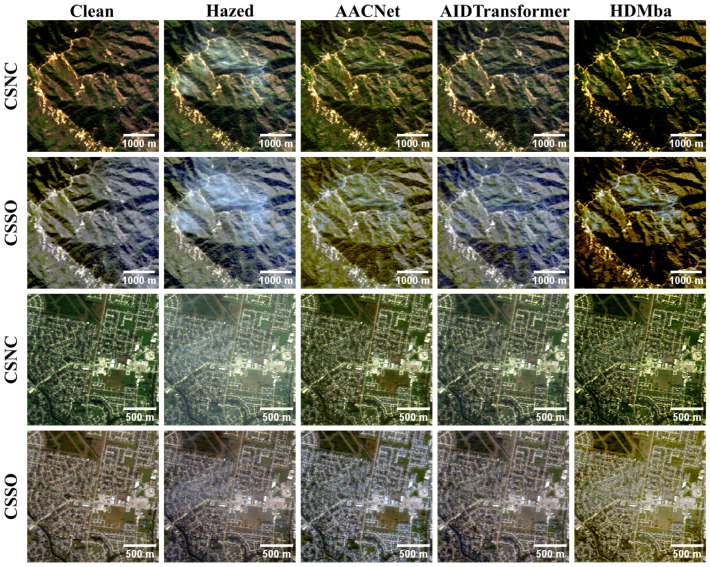
RGB visualization comparison of HS dehazing methods trained on the RSyntHyperPDID synthetic dataset and tested on the RRealHyperPDID real-world dataset.

**Figure 14:**
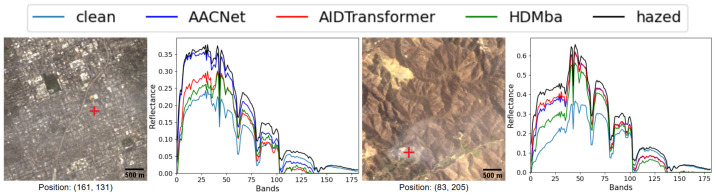
Spectral reconstruction performance of HS dehazing methods trained on the RSyntHyperPDID dataset. The red plus sign indicates the pixel location for which the spectral reconstruction is shown.

The qualitative analysis (see Figure 13) demonstrate that models trained on the RSyntHyperPDID effectively remove haze from real hazy images. This is corroborated by the spectral decomposition results (Figure 14). The quantitative results for the RSyntHyperPDID test set and the real-haze RRealHyperPDID dataset are presented in Table 9. According to the PSNR metric, all dehazing methods outperformed the “No dehazing” baseline, and paired statistical tests confirmed that these improvements are significant. In contrast, none of the methods exceeded the “No dehazing” baseline in terms of SSIM. For the SAM metric, only AACNet achieved a statistically significant improvement relative to the “No dehazing” baseline.

### 4.3. Downstream Task Quality Assessment Results

This section presents a DTQA comparison of dehazing methods based on their impact as preprocessing steps on the results of solving two downstream tasks: field delineation and land classification.

#### 4.3.1. Delineation-Based RGB Dehazing Quality Assessment

**Figure 15:**
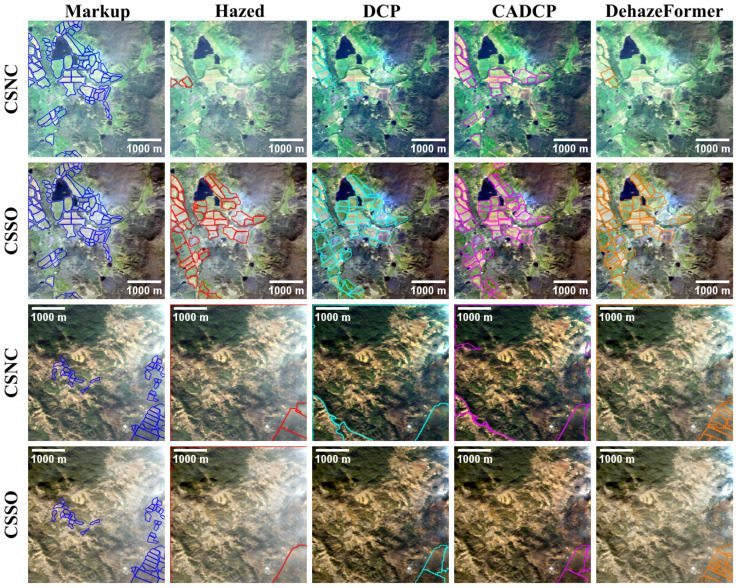
Results of applying the DA method to hazy and dehazed images generated using the CSNC and CSSO RGB synthesis methods. The colors of delineation masks indicate DA results applied to images processed by different dehazing methods.

Agricultural field delineation is performed using the DA method [39]. The results are summarized in Table 10 and illustrated in Figure 15. All tested dehazing methods improved delineation quality, with the DehazeFormer method demonstrating the largest performance gain.

#### 4.3.2. Classification-Based HS Dehazing Quality Assessment

**Figure 16:**
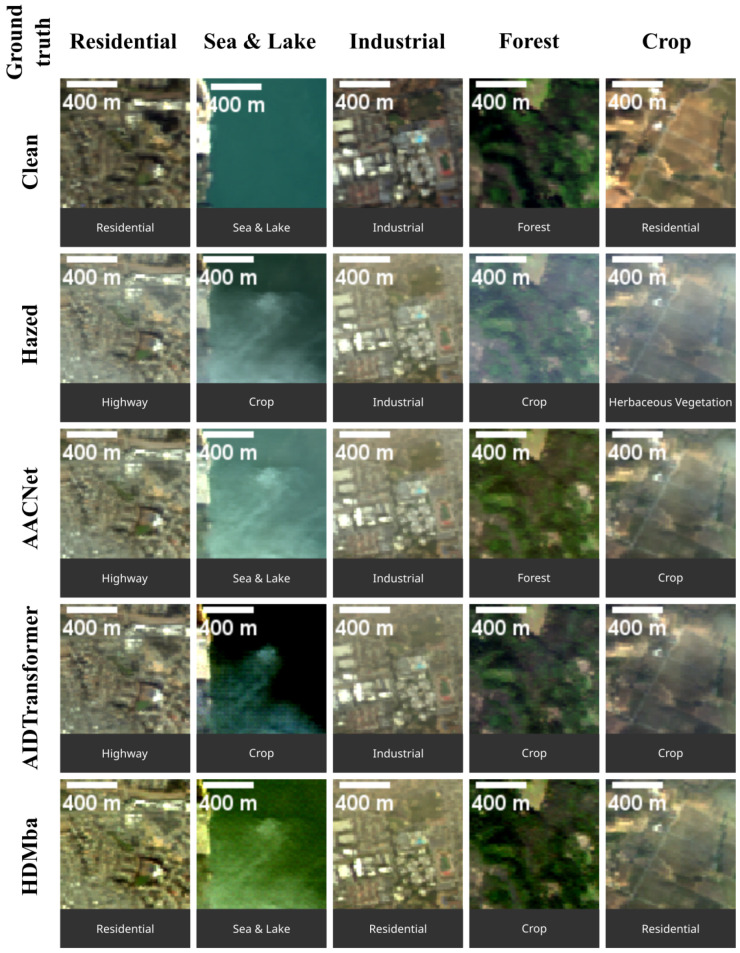
LULC classification results for hazy and dehazed images. The first row shows the ground truth class for each scene. The label below each image patch indicates the class predicted by the classification algorithm.

In this experiment, dehazing was first applied to the HS images, after which MS images were synthesized from the dehazed HSI outputs. The LULC classification algorithm was then evaluated on the resulting MS images. The classification accuracy is reported in Table 11. Examples of classification results are shown in Figure 16, visualized using CSNC RGB synthesis method.

The results in Table 11 indicate that dehazing generally improves LULC classification performance. Among the evaluated dehazing methods, AACNet achieved the highest accuracy, while HDMba showed no improvement over the hazy baseline, demonstrating the weakest performance.

## 5. Discussion

The limitations of the proposed RRealHyperPDID dataset include spatial and spectral inconsistencies between paired hazy and clean images. Such inconsistencies can compromise the reliability of FRQA metrics. Spatial misalignments arise from residual non-projective distortions, which remain even after co-registration procedures. A promising direction for future work is to develop more advanced alignment techniques to eliminate these residual distortions. Spectral discrepancies arise from the temporal gap between the acquisition of the clean and hazy images. These differences manifest as variations in illumination and surface properties. While closest scenes subset annotation and a nearest-clean-reference strategy for FRQA partially mitigates this issue, it cannot fully compensate for temporal spectral changes. Future studies should therefore focus on designing dedicated FRQA methods that evaluate dehazing performance using only the most stable surface elements. Aleatoric uncertainty quantification, as proposed in [3], could be employed to identify such stable elements.

Although the proposed benchmark datasets encompass diverse surface types—including mountains, urban areas, and agricultural regions—they are limited in climatic diversity. The absence of scenes representing other climatic zones is a notable constraint and an avenue for future expansion.

The proposed benchmark integrates the CCHS haze augmentation method, previously applied only to satellite MS imagery. In this work, we adapted CCHS for generating synthetic haze in airborne hyperspectral images. Using the proposed RRealHyperPDID, we show that CCHS produces physically realistic haze as models trained on synthetic data successfully generalize to real-world hazy conditions. This result validates both the universality of the CCHS method and the robustness of its underlying atmospheric scattering model, demonstrating its applicability to imagery captured at different altitudes, through varying atmospheric layers, and by sensors with distinct spectral responses.

In cases involving dense or fully opaque haze, HSIs alone may be insufficient for effective restoration, necessitating the use of auxiliary data. Such auxiliary information could include co-registered RGB images, HSIs, or SAR images of the same area. When auxiliary data are obtained at different times, a multi-temporal dehazing approach becomes essential. Notably, several RRealHyperPDID scenes contain multiple reference images, which can serve as auxiliary inputs for developing and evaluating multi-temporal dehazing methods. While SAR-assisted dehazing has been explored for MS imagery [47], HS dehazing with SAR guidance remains unexplored and represents a promising avenue for future research. A key prerequisite for this direction is robust HSI-SAR image alignment, a problem recently addressed in [48].

## 6. Conclusions

This work presents a comprehensive benchmark for hyperspectral remote sensing image dehazing (RSID) that integrates real and synthetic data, standardized evaluation protocols, and baseline implementations of state-of-the-art algorithms. The proposed benchmark includes data splits from the well-known HyperDehazing dataset, the proposed real-world test set (RRealHyperPDID) with paired hazy and haze-free hyperspectral images, and the proposed synthetic training set (RSyntHyperPDID) generated using the CCHS method. In addition, we provide an open-source evaluation suite that combines image quality and delineation-based metrics, establishing a unified framework for the assessment of dehazing performance in hyperspectral remote sensing.

The conducted experiments reveal several important insights. RGB-based RSID methods are capable of effectively compensating for real haze in remote sensing imagery. The neural network-based DehazeFormer, trained on synthetic data, demonstrates competitive performance, although classical model-driven approaches such as DCP and CADCP may still achieve superior results. The reliability of image quality metrics was found to depend strongly on the acquisition setup: in the case of CSSO imagery, common universal metrics such as the PSNR, SSIM, SAM, DISTS, and LPIPS showed poor correlation with expert evaluations, whereas for CSNC data, the PSNR, SSIM, and DISTS aligned well with expert judgment. Notably, PSNR remained consistent even in complex scenes where haze was not the sole source of change, establishing them as the recommended metric for evaluation. The development of metrics suitable for analyzing RGB images synthesized from HSIs using the CSSO method is an important direction for future research.

However, experiments on hyperspectral imagery revealed a clear domain gap: models trained exclusively on the synthetic HyperDehazing dataset failed to generalize to real haze conditions, even after applying spectral harmonization designed to mitigate channel mismatches. By contrast, HS dehazing models trained on the proposed RSyntHyperPDID dataset successfully remove real haze, indicating that RSyntHyperPDID provides a more representative and transferable basis for model training. Among the evaluated architectures, AIDTransformer achieved the highest PSNR, while AACNet demonstrated statistically significant improvements over “No dehazing” baseline in terms of both PSNR and SAM metrics.

The results also demonstrate the practical significance of dehazing as a preprocessing step. When RGB dehazing is applied prior to agricultural field delineation, DehazeFormer improved delineation accuracy across all types of RGB imagery, with little dependence on the specific imaging configuration. For land use and land cover classification, applying hyperspectral dehazing increases accuracy with AACNet, whereas HDMba provided no improvement compared to using hazy data.

Overall, the proposed benchmark provides a robust and reproducible foundation for advancing research in hyperspectral dehazing.

## Figures and Tables

**Table 1:** Comparison of the proposed datasets, RRealHyperPDID and RSyntHyperPDID, with existing remote sensing dehazing datasets.

Dataset	Size	Real/Synthetic	Paired	Resolution	Image Type
RRSHID [22]	3053	Real	Yes	256 × 256	RGB
RS-Haze [14]	51,300	Synthetic	Yes	512 × 512	RGB
HyperDehazing [24]	2000	Synthetic	Yes	512 × 512	Hyperspectral
HyperDehazing [24]	70	Real	No	512 × 512	Hyperspectral
HDD [25]	40	Real	No	512 × 512	Hyperspectral
RRealHyperPDID	110	Real	Yes	256 × 256	Hyperspectral
RSyntHyperPDID	2616	Synthetic	Yes	256 × 256	Hyperspectral

**Table 2:** Comparison of the DA agricultural field delineation method results for clean and hazy RGB images.

Data	DICE	DICE_obj_	Number of Images
clean (CSNC)	57.17	25.46	52
hazed (CSNC)	38.59	15.93	34
clean (CSSO)	51.24	22.8	52
hazed (CSSO)	42.61	20.93	34

**Table 3:** Results of various RGB dehazing methods applied to RRealHyperPDID images (CSNC RGB synthesis method). Entries show the mean values with 95% bootstrap confidence intervals (bias-corrected and accelerated, BCa). Bold indicates the best result, and underlined indicates the second-best.

Method	PSNR ↑	SSIM ↑	SAM ↓	DISTS ↓	LPIPS ↓	CD_proLab_ ↓
No dehazing	14.10 ± 0.54	0.605 ± 0.017	0.193 ± 0.019	0.231 ± 0.008	0.366 ± 0.013	0.160 ± 0.015
DCP [6]	16.81 ± 0.51	0.631 ± 0.020	0.234 ± 0.024	0.203 ± 0.007	0.359 ± 0.014	0.184 ± 0.016
CADCP [10]	**17.00 ± 0.51**	**0.635 ± 0.020**	0.217 ± 0.023	**0.198 ± 0.007**	**0.352 ± 0.014**	0.171 ± 0.015
DehazeFormer [14]	15.56 ± 0.49	0.631 ± 0.017	**0.180 ± 0.020**	0.208 ± 0.007	**0.352 ± 0.013**	**0.147 ± 0.015**

Arrows indicate: ↑ higher is better, ↓ lower is better.

**Table 4:** Results of various RGB dehazing methods applied to RRealHyperPDID images (CSSO RGB synthesis method). Entries show the mean values with 95% bootstrap confidence intervals (bias-corrected and accelerated, BCa). Bold indicates the best result, and underlined indicates the second-best.

Method	PSNR ↑	SSIM ↑	SAM ↓	DIST ↓	LPIPS ↓	CD_proLab_ ↓
No dehazing	16.71 ± 0.52	0.655 ± 0.016	**0.142 ± 0.013**	0.215 ± 0.009	0.378 ± 0.015	**0.130 ± 0.011**
DCP [6]	15.48 ± 0.46	0.602 ± 0.015	0.214 ± 0.015	0.216 ± 0.007	0.383 ± 0.015	0.178 ± 0.012
CADCP [10]	15.53 ± 0.45	0.603 ± 0.015	0.209 ± 0.014	0.215 ± 0.007	0.381 ± 0.015	0.175 ± 0.012
DehazeFormer [14]	**17.42 ± 0.50**	**0.657 ± 0.016**	0.155 ± 0.014	**0.198 ± 0.008**	**0.366 ± 0.016**	0.137 ± 0.013

Arrows indicate: ↑ higher is better, ↓ lower is better.

**Table 5:** Expert-based scores of RGB dehazing methods on the RRealHyperPDID dataset.

Method	CSNC	CSSO
No dehazing	0.0005	0.0007
DCP	0.3068	0.3558
CADCP	0.6762	0.6027
DehazeFormer	0.0165	0.0408

**Table 6:** Kendall correlation coefficients between metric-based and expert rankings of dehazing algorithms, shown separately for close and other scenes of RRealHyperPDID.

Close Scenes						Other Scenes				
PSNR	SSIM	SAM	DISTS	LPIPS		PSNR	SSIM	SAM	DISTS	LPIPS
					CSNC					
0.91	0.91	-	1	-		1	-	−0.33	-	−0.18
					CSSO					
−0.60	−0.60	−0.80	0.18	-		−0.55	−0.91	−0.91	-	−0.55

**Table 7:** Reproduction of neural network HSI dehazing results on the HyperDehazing test set. Results for hazy images were not provided in [18].

	[18] Results			Our Results		
Method	PSNR ↑	SSIM ↑	SAM ↓	PSNR ↑	SSIM ↑	SAM ↓
No dehazing	-	-	-	32.81	0.9513	0.0348
AACNet [23]	37.43	0.9734	0.0425	40.44	0.9572	0.0189
AIDTransformer [15]	35.47	0.9723	0.0401	38.13	0.9553	0.0262
HDMba [18]	38.13	0.9763	0.0382	36.16	0.9525	0.0352

Arrows indicate: ↑ higher is better, ↓ lower is better.

**Table 8:** Results of HS dehazing methods on the RRealHyperPDID dataset. Models were trained on the HyperDehazing training set. Entries show the mean values with 95% bootstrap confidence intervals (bias-corrected and accelerated, BCa). Bold indicates the best result, and underlined indicates the second-best.

Method	PSNR ↑	SSIM ↑	SAM ↓
No dehazing	**25.49 ± 0.73**	**0.685 ± 0.022**	**0.196 ± 0.016**
AACNet [23]	19.00 ± 0.52	0.517 ± 0.024	0.364 ± 0.021
AIDTransformer [15]	25.16 ± 0.73	0.682 ± 0.023	0.202 ± 0.017
HDMba [18]	22.19 ± 0.58	0.616 ± 0.025	0.280 ± 0.019

Arrows indicate: ↑ higher is better, ↓ lower is better.

**Table 9:** Results of neural network-based HS dehazing methods on the validation subset of the synthetic-haze RSyntHyperPDID and real-haze RRealHyperPDID. Models were trained on the RSyntHyperPDID training set. Entries show the mean values with 95% bootstrap confidence intervals (bias-corrected and accelerated, BCa). Bold indicates the best result, and underlined indicates the second-best.

	RSyntHyperPDID Test			RRealHyperPDID		
Method	PSNR ↑	SSIM ↑	SAM ↓	PSNR ↑	SSIM ↑	SAM ↓
No dehazing	22.61 ± 0.55	0.911 ± 0.009	0.133 ± 0.006	26.45 ± 0.74	**0.695 ± 0.022**	0.200 ± 0.016
AACNet [23]	**34.19 ± 0.38**	**0.972 ± 0.002**	**0.048 ± 0.003**	27.82 ± 0.75	0.688 ± 0.021	**0.182 ± 0.019**
AIDTransformer [15]	30.54 ± 0.57	0.955 ± 0.005	0.064 ± 0.004	**28.09 ± 0.65**	0.628 ± 0.016	0.214 ± 0.027
HDMba [18]	27.90 ± 0.57	0.941 ± 0.007	0.086 ± 0.006	27.65 ± 0.65	0.583 ± 0.015	0.237 ± 0.030

Arrows indicate: ↑ higher is better, ↓ lower is better.

**Table 10:** Comparison of DA results for hazy and dehazed images processed with DCP, CADCP, and DehazeFormer algorithms.

	CSNC		CSSO	
Data	DICE ↑	DICE_obj_ ↑	DICE ↑	DICE_obj_ ↑
hazed	38.59	15.93	42.61	20.93
DCP [6]	43.16	18.01	47.22	22.88
CADCP [10]	45.95	19.36	47.35	21.45
DehazeFormer [14]	46.92	20.55	50.31	23.22
clean	57.17	25.46	51.24	22.8

Up arrow (↑) indicates that higher values are better.

**Table 11:** LULC classification results on MS images synthesized from the RRealHyperPDID dataset.

Method	Accuracy
clean	74.56%
hazed	56.29%
AIDTransformer	59.86%
AACNet	62.47%
HDMba	56.29%

## Data Availability

The proposed datasets are publicly available at https://huggingface.co/datasets/nikos74/RRealHyperPDID (accessed on 19 November 2025) for RRealHyperPDID and https://huggingface.co/datasets/nikos74/RSyntHyperPDID (accessed on 19 November 2025) for RSyntHyperPDID. Additional datasets analyzed in this study at https://github.com/RsAI-lab/HyperDehazing (accessed on 10 October 2025) for the HyperDehazing dataset and https://huggingface.co/datasets/lwCVer/RRSHID (accessed on 10 October 2025) for the RRSHID dataset. All source code, including neural network architectures, preprocessing scripts, training routines, and evaluation tools, is openly available at https://github.com/iitpvisionlab/hyperhazeoff (accessed on 19 November 2025).

## Abbreviations

The following abbreviations are used in this manuscript:

RRealHyperPDID	Remote Sensing Real-World Hyperspectral Paired Dehazing Image Dataset
RSyntHyperPDID	Remote Sensing Synthetic Hyperspectral Paired Dehazing Image Dataset
HSI	Hyperspectral image
RSID	Remote sensing image dehazing
DCP	Dark Channel Prior
CNN	Convolutional neural network
MS	Multispectral
RRSHID	Real-World Remote Sensing Hazy Image Dataset
DTQA	Downstream task quality assessment
IQA	Image quality assessment
AS	Atmospheric scattering
HTM	Haze transmission map
HHS	Hazy HSI Synthesis
CCHS	Channel Correlation-Based Haze Synthesis
FRIQA	Full-Reference IQA
NRIQA	No-Reference IQA
RMSE	Root Mean Square Error
PSNR	Peak Signal-To-Noise Ratio
SSIM	Structural Similarity Index
SAM	Spectral Angle Mapper
LPIPS	Learned Perceptual Image Patch Similarity
NDVI	Normalized Difference Vegetation Index
DOS	Dark Object Subtraction
SIFT	Scale-Invariant Feature Transform
RANSAC	Random Sample Consensus
CSNC	Color Synthesis Narrow Channels
CSSO	Color Synthesis Standard Observer
DA	Delineate Anything
LULC	Land Use and Land Cover
CADCP	Color Attenuation Dark Channel Prior
CAP	Color Attenuation Prior
MSE	Mean Squared Error
